# The impact of levodopa therapy-induced complications on quality of life in Parkinson’s disease patients in Singapore

**DOI:** 10.1038/s41598-019-45110-5

**Published:** 2019-06-25

**Authors:** Jonathan Wu, Ee-Chien Lim, Nivedita V. Nadkarni, Eng-King Tan, Prakash M. Kumar

**Affiliations:** 1 Duke‐NUS Medical School, Singapore, Singapore; 2 Department of Neurology, National Neuroscience Institute (SGH campus), Singapore, Singapore; 3 Centre of Quantitative Medicine, Duke‐NUS Medical School, Singapore, Singapore

**Keywords:** Quality of life, Quality of life, Parkinson's disease, Parkinson's disease

## Abstract

The objective of this study was to investigate the impact of levodopa therapy-induced complications on the quality of life (QoL) of Parkinson’s disease (PD) patients in Singapore over a 1-year follow-up period. 274 PD patients were prospectively recruited, of which 78 patients completed the follow-up. Patients were evaluated on: (1) motor symptoms, (2) non-motor symptoms, (3) levodopa therapy-induced complications and (4) QoL. Levodopa-induced complications including dyskinesia and OFF symptoms occurred in 13.5% and 55.9% of the study population, respectively. In patients who completed the 1-year follow-up, there was a trend suggestive of increasing dyskinesia duration, more disabling dyskinesia as well as longer, more sudden and unpredictable OFF periods. There was a significant decline in the overall QoL at follow-up, in particular, activities of daily living, emotional well-being, cognition and communication domains were the most affected. The multivariable analysis demonstrated that worsening of UPDRS IV total score over 1-year interval was associated with worsening in PDQ-Summary Index score (d = 0.671, p = 0.014). In conclusion, levodopa-induced complications had significant adverse impacts on QoL. This study substantiates the importance for clinicians to closely monitor and promptly manage levodopa therapy-induced complications that may arise in patients.

## Introduction

Parkinson’s disease (PD) is the second-most prevalent neurodegenerative disease in the world, with the average age of onset of approximately 60 years^1^. It is most consistently linked with aging, and the global burden of PD is expected to increase sharply in future as life expectancies increase and demographics change^2^.

It is largely defined by the cardinal symptoms of resting tremor, bradykinesia, rigidity, and impaired postural reflexes and levodopa remains as the gold standard treatment for these symptoms for several decades^3^. However, long-term usage of levodopa can give rise to complications, with the incidence rate and severity of those complications correlated with increased cumulative dosage^3,4^.

Levodopa therapy-induced complications can be characterized by: (1) dyskinesia and (2) fluctuations in medication effectiveness (i.e. ON-OFF timings). The Unified Parkinson’s Disease Rating Scale part IV (UPDRS IV) was developed to measure complications of levodopa therapy and is based upon patients’ subjective reporting of their experienced symptoms^5^.

At present, there is no cure for PD. Assessing the patient’s quality of life (QoL) is therefore particularly helpful in allowing clinicians to determine the effectiveness of treatment^6^. As such, the primary goal of PD management focuses on treating the symptoms that have significant impact on the patient’s QoL.

The primary aim of this study was to determine the impact of levodopa therapy-induced complications on the quality of life of PD patients over a 1-year follow-up period.

## Results

### Demographics and clinical characteristics

The baseline demographics of patients who completed versus those who did not complete the 1-year follow-up were comparable (Table 1). There was significant difference between the baseline and follow-up visit for patients who completed 1-year follow-up for mean/median LEDD; PDQ-SI; the PDQ domains for ADL, emotional well-being, cognitive impairment, and communication; S&E ADL; NMSS; and total UPDRS III scores (Table 2).

**Table 1:** Baseline characteristics of PD patients.

Mean (SD), Median (IQR) or Frequency (%)			
Characteristics	Overall (n = 274)	Completed 1-year follow-up (n = 78)	Did not complete 1-year follow-up (n = 196)
Age in years	66.9 (9.01)	67.3 (8.57)	67.3 (8.93)
Male gender	167 (60.9)	52 (66.7)	115 (58.7)
Chinese ethnicity	235 (85.8)	73 (93.6)	162 (82.7)
Unemployed status	204 (74.5)	58 (74.4)	146 (74.5)
Lower than GCE O level of education	206 (75.2)	62 (79.5)	144 (73.5)
Disease duration in days	1989 (1509)	1971 (1463)	2125 (1708)
Age of onset in years	62 (55, 69)	62 (56, 67)	62 (54, 69)
Onset before age 50	29 (10.6)	7 (9.0)	22 (11.2)
Age at diagnosis in years	63 (56, 70)	63 (56.75, 68.25)	63 (56, 70)
No family history of PD	249 (90.9)	70 (89.7)	179 (93.4)
UPDRS IV total score	2.00 (0, 3.00)	2.00 (0, 4.00)	2.00 (0, 3.00)

Abbreviation - SD: Standard Deviation; IQR: Inter Quartile Range; UPDRS IV: Unified Parkinson’s Disease Rating Scale Part IV.

**Table 2:** Characteristic at baseline and 1-year follow-up.

Study Results	Mean (SD), Median (IQR) or Frequency (%)		Wilcoxon signed rank test
	Baseline (n = 78)	1 –year follow-up (n = 78)	
UPDRS IV total score	2.00 (0, 4.00)	3.00 (0.75, 4.00)	0.206
UPDRS IV Domain A score: Dyskinesia	0 (0, 0)	0 (0, 1.00)	0.030
UPDRS IV Domain B score: OFF symptoms	2.00 (0, 2.00)	2.00 (0, 3.00)	0.291
LEDD (mg)	300 (150.00, 536.50)	350.00 (200.00, 632.25)	0.000
PDQ-39-SI (%)	16.56 (9.93, 25.83)	23.15 (12.49, 33.75)	0.000
• Domain 1 - mobility	20.00 (5.00, 47.50)	28.75 (11.88, 53.12)	0.060
• Domain 2 – ADL	8.33 (0, 25.00)	20.83 (8.33, 41.67)	0.000
• Domain 3 - emotion	16.67 (4.17, 29.17)	25.00 (12.50, 33.33)	0.005
• Domain 4 – stigma	0 (0, 32.81)	0 (0, 31.25)	0.567
• Domain 5 – social	0 (0, 0)	0 (0, 25.00)	0.063
• Domain 6 – cognitive	18.75 (6.25, 37.50)	31.25 (0, 50.00)	0.001
• Domain 7 – communication	0 (0, 33.33)	16.67 (0, 33.33)	0.015
• Domain 8 – discomfort	25.00 (0, 41.67)	25.00 (14.58, 50.00)	0.068
S&E ADL score (%)	90.00 (80.00, 100.00)	90.00 (80.00, 90.00)	0.013
ECAQ score	10.00 (9.00, 10.00)	9.00 (9.00, 10.00)	0.378
NMSS total score	28.00 (15.75, 47.50)	43.50 (22.00, 60.00)	0.000
H&Y staging (≥3)	12 (15.4)	23 (29.5)	0.008
UPDRS III total score	23.55 (13.24)	29.85 (15.94)	0.000

Abbreviation – IQR: Inter Quartile Range; UPDRS IV: Unified Parkinson’s Disease Rating Scale Part IV; LEDD: Levodopa Equivalent Daily Dose; PDQ-39-SI: Parkinson’s Disease Questionnaire-39-Summary Index; S&E ADL: Schwab and England Activities of Daily Living Scale; ECAQ: Elderly Cognitive Assessment Questionnaire; NMSS: Non-Motor Symptoms Scale; H&Y: Hoehn and Yahr staging; UPDRS III: Unified Parkinson’s Disease Rating Scale Part III (motor).

### UPDRS IV results - Domain A (Dyskinesia)

In our overall patient sample, dyskinesia was the least common levodopa therapy-induced complication. At baseline, only 13.5% of the patients experienced dyskinesias during their waking hours. Most of the dyskinesia experienced was rated as not disabling (91.2%) and not painful (96.7%). A small proportion also experienced early morning dystonia (8.0%).

At 1-year follow-up, there was a trend suggestive of increasing dyskinesia duration (from 5.1% to 16.7%, 1.3% to 2.6%, and 1.3% to 2.3% for 1–25%, 26–50%, and 51–75% of their waking day, respectively), and more disabling (from 93.6% to 88.5% not disabling, 5.1% to 9.0% mildly disabling, and 1.3% to 2.6% as moderately disabling). An increasing proportion also experienced early morning dystonia (from 11.5% to 21.8%).

### UPDRS IV results - Domain B (Clinical fluctuations)

Clinical fluctuations were the most frequent levodopa-induced complications. At baseline for the entire sample, 39.1% had OFF periods not more than 25% of the waking day, while 14.2% and 2.6% reported experiencing them for 25–50% and 51–75% of their waking day, respectively. A majority had gradual (98.2%) and predictable (54.0%) OFF periods.

For the 1-year follow-up group, there was a shift in responses to having longer duration (from 41.0% to 32.1%, 11.5% to 21.8%, and 5.1% to 7.1% for 1–25%, 26–50%, and 51–75% of the patient’s waking day, respectively), more sudden onset (from 0% to 6.4%), and unpredictable (from 1.3% to 3.3%) OFF periods.

### Univariate analysis

The univariate analysis showed age, gender, employment status, disease duration, ECAQ, subsequent visit, UPDRS IV total and domain scores, LEDD, NMSS, UPDRS III, H&Y staging and S&E ADL score were statistically significant and individually predictive of change in PDQ-SI score from baseline to 1-year follow up.

### Multivariable analysis

The multivariable analysis (Table 3) found that change in UPDRS IV total score was associated with a medium effect change in PDQ-SI (d = 0.671, p = 0.014). The other factors with smaller effect but stronger statistical significance included female gender (d = 0.374, p < 0.001), NMSS total score (d = 0.305, p < 0.001) and UPDRS III total score (d = 0.179, p < 0.001).

**Table 3:** Linear mixed model analysis: PDQ-SI as a function of potential clinical factors.

Covariate	Multivariable analysis		
	Parameter Estimate	95% CI	p-value
Age in years	—	—	—
Age of onset in years	—	—	—
Age at diagnosis in years	—	—	—
Male gender	−0.374	(−0.184, −0.565)	0.000
Unemployed status	—	—	—
Lower than GCE O level of education	—	—	—
Onset before age 50	—	—	—
Disease duration in days	—	—	—
ECAQ score	—	—	—
1-year follow-up visit	—	—	—
UPDRS IV total score	0.671	(0.129, 1.200)	0.014
LEDD in mg	—	—	—
NMSS total score	0.305	(0.257, 0.352)	0.000
H&Y staging (≥3)	0.358	(0.185, 0.637)	0.012
UPDRS III total score	0.179	(0.100, 0.258)	0.000
S&E ADL	—	—	—

Abbreviation - ECAQ: Elderly Cognitive Assessment Questionnaire; UPDRS IV: Unified Parkinson’s Disease Rating Scale Part IV; LEDD: Levodopa Equivalent Daily Dose; NMSS: Non-Motor Symptoms Scale; H&Y: Hoehn and Yahr staging; UPDRS III: Unified Parkinson’s Disease Rating Scale Part III (motor); S&E ADL: Schwab and England Activities of Daily Living Scale.

## Discussion

To the best of our knowledge, this short-term longitudinal study is one of the first examining the impact of levodopa therapy-induced complications on QoL in PD patients in a multi-ethnic Asian population like Singapore’s. The majority of other studies have been non-longitudinal and conducted on non-Asian populations^7–10^, while relatively few examined the determinants of QoL in PD patients in Chinese or predominantly Chinese populations^11,12^.

Our study results have shown that levodopa therapy-induced complications have an impact on patients’ QoL and is consistent with other studies conducted in both Asian and Caucasian populations^7–9,12^. In addition, this study has shown some evidence that suggest deterioration of these complications had a medium effect on deterioration of the overall QoL in PD patients for the 1-year follow-up interval. This finding, to our knowledge, has not been published elsewhere and it suggests that treatment related complications are important to be explored during follow-ups of PD patients.

Previous studies had inconsistent results regarding gender, age, and educational attainment as risk factors for worsening QoL^10–12^. This may possibly be due to cultural or ethnic differences across population groups. In our study, apart from levodopa therapy-induced complications, other lesser predictive factors included female gender, NMSS total score and UPDRS III total score. The findings suggest that patients with these factors may be anticipated to have worsening of QoL over time if their symptoms are not managed appropriately.

Longer disease duration is known to be associated with worsening QoL and this was also demonstrated in our study^9,13–15^. While disease duration is a well-studied clinical variable, fewer studies examined the impact of age of onset on QoL^12^. Our study, as well as other studies, did not find age of onset to have any significant correlation with QoL^12,16,17^.

In this study, patients’ perceived QoL – one of the key measures of effective management of PD – declined significantly even within a short 1-year period. The PDQ domain scores for ADL, emotional well-being, cognitive impairment and communication increased the most from baseline to 1-year follow-up. Therefore, when addressing patients’ complaints, clinicians may prioritize addressing treatment complications related to these domains.

Our study has several limitations. Firstly, the sample group was rather homogenous with most PD patients in the mild to moderate stages (H&Y stage < 3), therefore it is likely that the more severe motor complications were not captured in this population group. This may limit the generalizability of our results to those patients with more advanced disease stages, who are likely to have more severe motor complications. Secondly, patients who were cognitively impaired and/or unable to verbalize were excluded from the study, as the format of the study assessments was reliant on patients’ ability to recall and communicate the symptoms that they had experienced. Hence, those with poor communicative ability or cognitive issues could be under-represented in our study. In addition, less than a third of the participants completed the 1-year follow-up visit. However, this is an on-going study and patients are still continuously being assessed and followed-up. Lastly, this study lacked normal controls when evaluating differences in PDQ-SI score. However, the main focus of the study was on the longitudinal evaluation of QoL deterioration in PD patients.

In conclusion, our study has shown that levodopa therapy-induced complications have a significant adverse impact on QoL, in particular, the ADL, emotional well-being, cognition and communication domains in PD patients. Worsening of these complications had a medium effect on the worsening of the overall QoL for the 1-year follow-up interval. This substantiates the importance for clinicians to closely monitor and promptly manage the levodopa therapy-induced complications that may arise in PD patients. Further studies that involve developing strategies for effective management, delaying the onset, or even possibly preventing these complications will be valuable to explore.

## Methods

This was a mono-center, observational, short-term longitudinal study.

### Inclusion criteria

PD patients, diagnosed according to the UK PD Brain Bank criteria, were prospectively recruited from Singapore General Hospital neurology and movement disorders clinics^18^. A total of 274 patients were enrolled, of which 78 patients were reassessed at 1-year follow-up.

### Exclusion criteria

Patients with significant cognitive impairment as defined by an Elderly Cognitive Assessment Questionnaire (ECAQ) score of 5 points or less were excluded to avoid unreliable responses for the many subjective patient-dependent scores. Similarly, patients with significant speech dysfunction who were not able to verbalize appropriately were excluded. Patients with chronic debilitating conditions (e.g. severe heart failure, liver failure, renal failure requiring dialysis, or other terminal illness) were also excluded.

The Singhealth Centralized Institutional Review Board SCIRB approved the study protocol. All methods were carried out in accordance with SCIRB guidelines and regulations, including informed consent from all subjects.

### Assessments

Demographic data, including age, gender, educational status, family history, ages of onset and diagnosis, and disease duration were obtained from all recruited patients.

Patient QoL was measured by the 39-item Parkinson’s Disease Questionnaire (PDQ-39), the most widely used disease specific health-related QoL instrument in clinical practice and research^6,19,20^. It incorporates eight disease domains: mobility (10 items), ADL (6 items), emotional well-being (6 items), stigma (4 items), social support (3 items), cognition (4 items), communication (3 items), and bodily discomfort (3 items). Responses were scored from a scale of 0 (never) to 4 (always). Each domain score was transformed into a 0–100 percentage scale by summing the domain items’ raw scores, dividing by the maximum possible raw score, and then multiplying by 100. A PDQ Summary Index (PDQ-SI) was obtained by calculating the mean of the eight domain scores. Higher scores reflected a poorer patient-rated QoL^6^.

Levodopa therapy-induced complications were assessed with the UPDRS IV questionnaire – a validated instrument that evaluates complications of levodopa therapy, and divides answers into 2 domains: domain A: dyskinesia (4 questions) and domain B: clinical fluctuations (4 questions)^5^.

All patients had their PD medication regime converted into the levodopa equivalent daily dose (LEDD) via the standardized formulae: ([levodopa (mg)] + [controlled release levodopa (mg) × 0.75] + [levodopa doses taken together with entacapone (mg) × 1.33] + [pramipexole (mg) × 100] + [ropinirole (mg) × 20] + [rotigotine (mg) × 30] + [piribedil (mg) × 1] + [bromocriptine (mg) × 10] + [selegiline (mg) × 10])^21^.

Non-motor symptoms were assessed using the Non-Motor Symptoms Scale (NMSS)^22^.

Other assessments included (1) Unified Parkinson’s Disease Rating Scale Part III (UPDRS III)^23^, (2) modified Hoehn and Yahr (H&Y) staging scale^24^ and (3) the Schwab and England activities of daily living (S&E ADL)^25^.

Assessments were conducted face-to-face on the same day of visit to the specialist clinic by movement disorder neurologists & trained research associates. The interval between baseline and follow-up visit ranged from 9 to 18 months. All subjects were provided with symptomatic treatment (pharmacological or non-pharmacological) as deemed necessary by the treating movement disorders specialist.

### Statistical analysis

Continuous data were summarized as mean (SD) or median (IQR), while categorical data was expressed in terms of frequency. Differences between time points were evaluated using the non-parametric Wilcoxon signed-rank test. A p-value of <0.05 was set to be significant.

A linear mixed effects model with subject as a random effect was used to assess the change in PDQ-SI as a function of UPDRS IV over time (2 time points) and adjusting for other factors such as LEDD, UPDRS III score, NMSS score, S&E ADL score, H&Y score, age, disease duration, gender, employment status, etc. Model diagnostics was used to test model adequacy.

## Data Availability

The datasets analyzed during the current study are available from the corresponding author on reasonable request.
